# Effect of Replacing Conventional Corn with Corn Containing Thermostable α-Amylase Enzyme (AMY797E) in Standard and Low-Energy Diets in Laying Hens

**DOI:** 10.3390/ani16040582

**Published:** 2026-02-12

**Authors:** Deependra Paneru, Dima White, Milan Sharma, John Gonzalez, Woo Kim

**Affiliations:** 1 Department of Poultry Science, University of Georgia, Athens, GA 30602, USA; dpaneru@uga.edu; 2 Global Animal Products, Amarillo, TX 79118, USA; dwhite@globalanimalproducts.com; 3 Department of Food Science, Cornell University, Ithaca, NY 14853, USA; mks279@cornell.edu; 4 Department of Animal and Dairy Science, University of Georgia, Athens, GA 30602, USA; johngonz@uga.edu

**Keywords:** Enogen corn, α-amylase, energy density, laying hens, bone microarchitecture

## Abstract

In laying hens, the undigested starch in conventional corn can reduce the efficiency of their diet. Therefore, the current study evaluated the effects of replacing conventional corn with a corn hybrid expressing thermostable α-amylase (Enogen corn) in standard and energy-deficient diets in laying hens. A total of 320 White Leghorn hens were assigned to four dietary treatments in a 2 × 2 factorial design of corn type (conventional and Enogen) and energy density (standard and 200 kcal/kg reduction) from 18–45 weeks of age. Enogen corn diet improved overall feed efficiency by 8% (*p* < 0.001) and increased egg production by 6–10% (*p* < 0.05) during 35–45 weeks compared to conventional corn diet. Yolk weight was also improved by Enogen corn at 35 and 45 weeks (*p* < 0.05), with no significant effect on body weight and body composition (*p* > 0.05). Reducing dietary energy led to higher feed intake during 36–45 weeks (*p* = 0.017), worsened overall feed efficiency (*p* = 0.030), and decreased cortical bone mineral density (*p* = 0.035). In conclusion, replacing conventional corn with Enogen corn improved feed efficiency and egg production, whereas a 200 kcal/kg energy reduction triggered compensatory intake and impaired cortical bone quality.

## Simple Summary

Egg producers always seek to maximize the conversion of feed into eggs by improving feed ingredients. A major component of layer feed is corn, which contains starch that is not always fully digested. This inefficiency can lead to wasted energy and reduced performance. We tested whether replacing conventional corn with Enogen corn (a hybrid that expresses a thermostable starch-digesting enzyme) could improve the performance of laying hens. A total of 320 hens were fed either conventional or Enogen corn under two energy levels (standard or reduced) from 18 to 45 weeks of age. We measured egg production, feed efficiency, body composition, and bone health. Hens fed Enogen corn used less feed per dozen eggs by about 8% and laid 6–10% more eggs during 35–45 weeks of age, without changes in body weight, body fat, and muscle. Lowering dietary energy reduced the feed efficiency by about 5% and increased the porosity of cortical bone. Enogen corn did not prevent these bone changes. However, Enogen corn improved feed efficiency and egg production. Therefore, replacing conventional corn with Enogen corn can help producers lower feed costs in laying hen diets, but diets must be supplied with adequate energy to protect bone health.

## 1. Introduction

Starch is the most abundant nutrient in poultry diets and the major source of metabolizable energy (ME) [1]. The main dietary source of starch is cereal grains, with corn being the predominant ingredient in most commercial poultry feeds [2]. Although starch digestion in chickens is generally efficient, some studies suggests that a measurable proportion of dietary starch can escape complete hydrolysis in the small intestine [2,3]. The extent of starch digestion depends largely on the molecular structure of the starch granule. Starch is composed of two polymers: amylose, a linear chain of α-1,4 linked glucose units, and amylopectin, a highly branched molecule with both α-1,4 and α-1,6 linkages. Amylopectin is more accessible to enzymatic hydrolysis by pancreatic α-amylase, whereas amylose tends to form compact crystalline regions that are less digestible [4]. Thus, cereals with a lower amylose-to-amylopectin ratio generally have higher starch digestibility in poultry [5]. In corn, starch typically consists of about 25% amylose and 75% amylopectin, although this ratio can vary among cultivars [6]. The amylopectin fraction of corn starch is efficiently digested, whereas the amylose fraction is more resistant to enzymatic hydrolysis by endogenous amylases [7]. Another limitation is that chickens have limited capacity for microbial fermentation in the hindgut compared with mammals, so resistant starch that bypasses small intestinal digestion is largely lost rather than recovered through fermentation [8].

To overcome this limitation, exogenous carbohydrase enzymes such as α-amylase are often included in poultry feeds to improve starch utilization [9]. A major practical limitation of this approach is the poor heat stability of most enzyme additives during feed pelleting [10,11]. Pelleting involves conditioning the mash diet with steam at 70–90 °C, which can denature enzymes and drastically reduce their activity [11]. The potential solution to this problem is either the development of strategies to protect enzymes by post-pellet application or using thermostable coatings and the introduction of intrinsically thermostable enzyme variants that can withstand feed processing. Building on these principles, a novel strategy has been introduced through transgenic corn engineered to express a thermostable α-amylase (AMY797E) in the endosperm [12,13]. Originally developed to facilitate starch breakdown during high-temperature ethanol production, this grain contains an intrinsic amylase functionally similar to bacterial thermostable α-amylases used in commercial enzyme products [14]. The AMY797E enzyme remains inactive in raw grain but becomes active under pelleting and digestive conditions [12], eliminating the need for external enzyme supplementation.

Feeding trials with thermostable α-amylase corn in pigs and cattle have reported mixed results, with some studies showing improvements in growth and feed efficiency, while others report no measurable benefits, which could be due to species or diet-dependent effects [15,16,17]. Monogastric animals such as pigs, which rely heavily on small intestinal digestion, may benefit more when diets are high in resistant starch or contain ingredients with lower starch digestibility, whereas in diets already formulated with highly digestible starch sources, the added enzymatic activity provides limited advantage [18]. However, ruminants such as cattle rely mainly on ruminal microbial fermentation, which largely degrades starch and makes additional intrinsic or supplemental amylases relatively redundant [19]. Even within a species, diet composition has variable effects as improvements are more apparent under nutrient-restricted or lower-energy diets, but negligible when animals are fed energy-dense rations that already meet or exceed requirements [20]. In poultry, research on AMY797E corn is limited and data are still emerging. A study in broilers reported slightly higher feed intake and body weight with AMY797E corn but no clear improvements in feed conversion ratio (FCR) compared to conventional corn [21]. However, studies supplementing conventional diets with exogenous amylase in chickens have often shown performance improvements, especially in low ingredient quality or nutrient-restricted diets [22,23,24]. These results suggest that the response to amylase supplementation, whether through additives or transgenic grain, depends on factors such as diet composition, bird age, and gut microbial activity.

Given the limited research in laying hens and the inconsistent results across species, the present study was designed to evaluate the effects of replacing conventional corn with thermostable α-amylase (AMY797E) corn in layer diets formulated at either standard or reduced energy levels. The objective of the current study was to determine whether intrinsic amylase expression in the corn can improve laying performance, body composition, egg quality and bone health in laying hens under energy-restricted conditions where starch digestibility may be more limiting.

## 2. Materials and Methods

### 2.1. Birds and Experimental Design

A 2 × 2 factorial design was used to evaluate the effects of energy density (standard and reduced) and corn type (conventional and Enogen) on laying hen performance and bone health. A total of 320 White Leghorn pullets sourced from Hy-Line North America (Mansfield, GA, USA) were randomly assigned at hatch to four dietary treatments based on the combination of energy density and corn type, with 10 replicates per treatment and 8 birds per replicate. Results from the pullet phase are reported in our previous study [25], and the same birds were maintained on their respective treatments through the laying period with consistent dietary treatments from rearing to production.

At 18 weeks of age, hens were transferred to individual cages (48 × 32 × 40 cm^3^; 1536 cm^2^/hen) with a nipple drinker and a linear feeder until 45 weeks of age. Environmental conditions followed breeder guidelines, including a 16L:8D photoperiod and a temperature of 22 °C [26]. Diets were formulated as corn–soybean meal mash to meet or exceed nutrient requirements based on breeder guideline [26] except for dietary energy and provided in two phases: peaking (18–37 weeks) and layer 2 (38–45 weeks). The four dietary treatments were (1) standard energy + conventional corn, (2) standard energy + Enogen corn (thermostable α-amylase, AMY797E), (3) reduced energy + conventional corn and (4) reduced energy + Enogen Corn. Enogen corn completely replaced conventional corn in the relevant treatments. The reduced-energy diets were formulated with a 200 kcal/kg decrease in ME compared to standard-energy diets and were achieved by replacing soybean oil with inert sand. This level of energy reduction was chosen based on previous studies demonstrating that it produces measurable differences in performance without inducing severe nutritional deficiencies [27,28]. The analyzed composition of conventional and Enogen corn is presented in Table 1, and diet formulations are presented in Table 2 and Table 3.

Nutrient composition of conventional and Enogen corn were determined using the rooster assay [29] and previously reported in our pullet phase experiment [25].

### 2.2. Data Collection

Body weight (BW) and feed intake were recorded at 18, 25, 35, and 45 weeks of age. Egg production was monitored daily, and hen-day egg production (HDEP) as well as egg quality were measured every 5 weeks from 25 to 45 weeks. At 45 weeks, hens were euthanized by cervical dislocation for whole-body composition analysis and femur microarchitecture analysis.

### 2.3. Hen-Day Egg Production and Egg Quality Analysis

The HDEP was calculated at 5-week intervals (25, 30, 35, 40, and 45 weeks of age) using the formula:

(1) $$HDEP\left(\%\right)=\frac{Total\;eggs\;laid}{Total\;hen\;days}\times 100$$

where hen-days were defined as the product of the number of days in the period and the number of hens alive during that period [30].

Egg quality parameters were measured at the same intervals using 3 randomly selected eggs per replicate pen (30 eggs/treatment). Eggs were weighed using a digital balance (OHAUS Corporation, Parsippany, NJ, USA), and specific gravity was determined by flotation in a graded series of NaCl solutions (1.070–1.090 in 0.005 increments) [31]. Eggs were then manually separated into albumen, yolk, and eggshell components [32]. Albumen height was measured using a digital height gauge (Technical Services and Supplies Ltd., York, UK), and yolk and albumen weights were recorded. After rinsing, eggshells were dried at room temperature for 72 h, weighed, and thickness was measured at 3 equatorial locations using a digital micrometer (AMES, Cranston, RI, USA). Component percentages (yolk, albumen, eggshell) were calculated relative to total egg weight. Haugh unit was calculated using the formula:

(2) $$Haugh\;Unit\;(HU)=100\times {log}_{10}[H+7.57-1.7W^{0.37}]$$

where H is albumen height (mm) and W is egg weight (g) [33].

### 2.4. Whole-Body Composition Using DEXA

Whole-body composition was measured using a Lunar Prodigy dual energy X-ray absorptiometry (DEXA) system (GE Healthcare, Boston, MA, USA) following the avian protocols [34]. Birds were euthanized by cervical dislocation and placed in chest-up position on the DEXA scanning bed [35]. Before each scanning session, the DEXA system was calibrated using a hydroxyapatite–aluminum phantom. Scans were performed with a radiation dose of 0.18 µGy, a scan speed of 2.5 mm/s, and a voxel resolution of 0.07 mm × 0.07 mm × 0.50 mm [36]. Acquired images were analyzed with enCORE software (ver. 12.20.023, GE Healthcare) with a small-animal algorithm. Regions of interest were manually defined to encompass the full body of each bird, and body composition parameters were assessed. Whole-body composition parameters measured included bone mineral density (BMD), bone mineral content (BMC), bone area, fat mass, muscle mass, and their corresponding percentages.

### 2.5. Microarchitectural Analysis of Femur Bone

Femur microarchitecture was analyzed using Skyscan 1275 micro-computed tomography (micro-CT; Bruker, Kontich, Belgium). Following euthanasia, femur bones were dissected, cleaned of residual soft tissue, and stored at −20 °C until further analyses [37]. Prior to scanning, bones were thawed at 4 °C for 24 h. Remaining soft tissues were removed, and each bone was individually wrapped in a wet cheesecloth to prevent dehydration during scanning, as previously described [38]. The micro-CT system was calibrated prior to scanning using the manufacturer’s protocol. An alignment test and flat-field correction were performed to ensure optimal signal uniformity and detector response. Two hydroxyapatite phantoms (diameter: 8 mm; densities: 0.25 and 0.75 g/cm^3^) were scanned to calibrate BMD measurements. After calibration, wrapped femurs were secured in low-density polyethylene tubes (50 mL) and mounted on the scanning stage. Scans were acquired under the following settings: X-ray source: 80 kV voltage, 125 µA current; filter: 0.5 mm aluminum to minimize beam hardening; pixel resolution: 25 µm isotropic; rotation step: 0.4° with 4-frame averaging per step; and scanning range: 180° with random movement to reduce ring artifacts [39].

**Figure 1:**
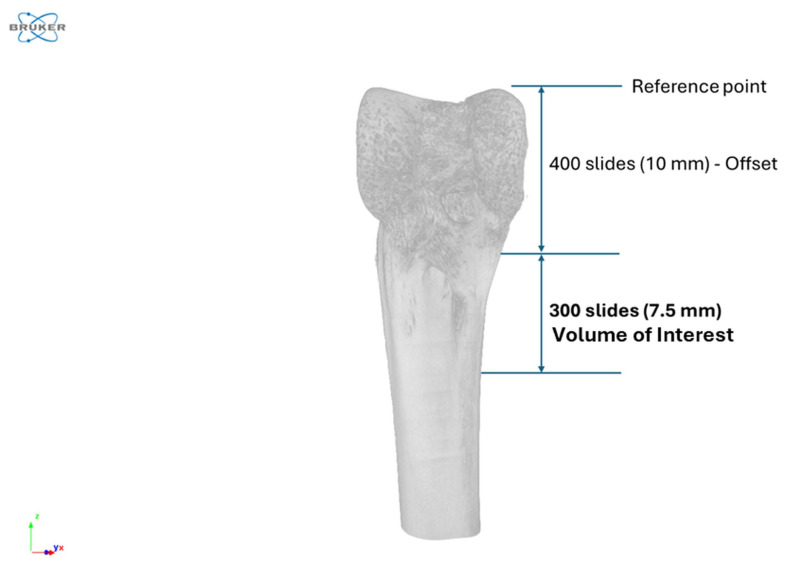
Selection of volume of interest (VOI) in the femur diaphysis for microarchitectural analysis. The VOI begins 400 slices (10 mm) below the top of the epiphyseal plate and spans 300 consecutive slices (7.5 mm) to analyze the cortical, trabecular, and medullary bone microarchitecture.

Two-dimensional (2D) projection images were reconstructed into three-dimensional (3D) volumes using NRecon software (v.1.7.4.2, Bruker) with a 35% beam-hardening correction to mitigate artifacts [40]. The 3D images were reoriented in Data Viewer software (v.1.5.6.2, Bruker) to standardize anatomical positioning. A volume of interest (VOI) was defined in the diaphysis region, starting 400 slices (10 mm) below the top of the epiphyseal plate, using CTAn software (v.1.20.8.0, Bruker). The VOI encompassed 300 consecutive slices (7.5 mm) to include the microarchitecture of cortical, trabecular, and medullary bone (Figure 1). Bone microarchitectural parameters were calculated using Batch Manager (BatMan) function in CTAn software.

### 2.6. Statistical Analysis

All statistical analyses were performed using JMP Pro software (version 18.0.2; JMP Statistical Discovery LLC, Cary, NC, USA). The experimental unit for all analyses was the pen. Data collected during the experiment were analyzed using a two-way ANOVA to evaluate the main effects of energy density, corn type, and their interaction. Normality of residuals was assessed using the Shapiro–Wilk test, and homogeneity of variances was verified using Levene’s test. For significant interaction effects (*p* < 0.05), post hoc comparisons were conducted using Tukey’s honest significant difference (HSD) test, whereas main effects were interpreted using Student’s *t*-tests. Results were reported as the least squares means with pooled standard error of mean (SEM). Statistical significance was defined at *p* < 0.05, and trends were noted at 0.05 ≤ *p* < 0.10 [38].

## 3. Results

### 3.1. Growth Performance

The effects of energy density and corn type on BW, body weight gain (BWG), feed intake, and FCR per dozen eggs of laying hens are presented in Table 4, Table 5, Table 6 and Table 7. Body weight was stable across treatments throughout the study. At weeks 25, 35, and 45 (Table 4), the energy density × corn type interaction was not observed (*p* > 0.05), and BW did not differ by energy density or corn type at any time point (*p* > 0.05).

Body weight gain also remained similar among treatments (Table 5). Across weeks 18–25, 26–35, 36–45, and overall (18–45), BWG was not affected by the interaction between energy density and corn type, and no main effects of energy density or corn type were observed (*p* > 0.05).

Feed intake responses differed by period (Table 6). During weeks 36–45, an energy density × corn type interaction was observed (*p* = 0.017), where hens fed the low-energy Enogen diet consumed more feed than those fed the standard-energy Enogen diet, while both conventional corn treatments were intermediate. However, feed intake did not differ among treatments during weeks 18–25, 26–35, or for the cumulative 18–45 period (*p* > 0.05).

For FCR per dozen eggs (Table 7), no significant interactions were observed between energy density and corn type at any period (*p* > 0.05). However, the main effect of corn type was significant for 26–35 weeks (*p* < 0.001), 36–45 weeks (*p* = 0.004), and the overall 18–45 weeks (*p* < 0.001). Hens fed Enogen corn showed lower FCR compared to those fed conventional corn during these periods. Additionally, the main effect of energy density was significant for 36–45 weeks (*p* = 0.004) and the overall 18–45 weeks (*p* = 0.030). Hens on low-energy diets had higher FCR compared to those on standard-energy diets.

### 3.2. Hen-Day Egg Production and Egg Quality

The effects of energy density and corn type on HDEP and egg quality parameters of laying hens are presented in Table 8, Table 9, Table 10, Table 11 and Table 12. At 25 weeks of age (Table 8), no significant interactions between energy density and corn type were observed for any egg quality parameter (*p* > 0.05). Energy density did not affect egg weight, yolk and albumen proportions, eggshell traits, and HDEP (*p* > 0.05); however, Haugh unit tended to be higher in hens fed the low-energy diet compared with the standard-energy diet (*p* = 0.068). Corn type significantly affected Haugh unit, with eggs from hens fed conventional corn showing higher HU values than those fed Enogen corn (*p* = 0.040) Table 8. Egg quality parameters of laying hens fed conventional corn or Enogen corn diets with standard or reduced energy at 25 weeks of age.

By 30 weeks (Table 9), no significant interactions between energy density and corn type were observed for any egg quality parameter (*p* > 0.05). Energy density alone did not affect egg weight, albumen weight, eggshell properties, HDEP, and Haugh unit (*p* > 0.05). However, corn type had a significant main effect on yolk weight, with hens fed conventional corn producing eggs with heavier yolks compared to those fed Enogen corn (*p* = 0.040). All other measures, including egg weight, albumen proportion, eggshell quality, and albumen quality, remained unaffected by corn type.

At 35 weeks of age (Table 10), no significant interactions between energy density and corn type were observed for any egg quality parameter (*p* > 0.05). Energy density alone did not significantly affect egg weight, yolk and albumen composition, or eggshell quality, although a trend toward lower HDEP was observed in the reduced-energy group (*p* = 0.070). However, corn type significantly affected yolk weight and hen-day egg production. Hens fed Enogen corn produced eggs with heavier yolks (*p* = 0.013) and achieved higher HDEP (*p* = 0.001) compared with hens fed conventional corn. Other parameters, including egg weight, albumen weight, specific gravity, and Haugh unit, were unaffected by corn type (*p* > 0.05).

At 40 weeks of age (Table 11), no significant interactions between energy density and corn type were observed for any egg quality parameter (*p* > 0.05). Energy density alone did not significantly affect egg weight, yolk weight, albumen weight, eggshell traits, or Haugh unit, although a trend toward reduced HDEP was observed in hens on low-energy diets compared with those on standard-energy diets (*p* = 0.055). However, corn type significantly affected yolk percentage and hen-day egg production. Hens fed Enogen corn produced eggs with a greater proportion of yolk (*p* = 0.043) and with higher HDEP (*p* = 0.002) compared with hens fed conventional corn. Other egg quality parameters, including eggshell thickness, specific gravity, and albumen quality were unaffected by corn type.

At 45 weeks of age (Table 12), several interactions between energy density and corn type were observed for albumen weight (*p* = 0.018), yolk percentage (*p* = 0.036), and albumen percentage (*p* = 0.010). Hens fed Enogen corn under standard-energy diets produced eggs with greater albumen weight, whereas those on low-energy Enogen diets had the highest yolk percentage and lowest albumen percentage. The main effects of corn type were significant for egg weight (*p* = 0.004), yolk weight (*p* = 0.026), and HDEP (*p* = 0.015), with Enogen corn consistently improving these parameters. A trend toward higher eggshell weight with Enogen corn (*p* = 0.059) was also observed. Energy density showed a near-significant effect on HDEP (*p* = 0.053), with standard-energy diets outperforming low-energy diets. Other parameters showed no significant differences (*p* > 0.05).

### 3.3. Whole Body Composition

The effects of energy density and corn type on body composition parameters of laying hens at 45 weeks of age are presented in Table 13. Body composition was not affected by dietary energy density, corn type, or their interaction (*p* > 0.05). The BMD, BMC and bone area were similar among treatments. Similarly, body fat percentage, fat weight, muscle percentage, muscle weight, and total fat + muscle weight, were similar among treatments.

### 3.4. Microarchitecture of Femur Bone

The effects of energy density and corn type on femur bone microarchitecture in laying hens at 45 weeks of age are presented in Table 14, Table 15, Table 16 and Table 17. For the total bone section (Table 14), no significant interaction effects between energy density and corn type were observed for BMD, BMC, tissue volume (TV), bone volume (BV), or bone volume fraction (BV/TV; *p* > 0.05). Similarly, no significant main effects of energy density or corn type were observed (*p* > 0.05).

In the cortical bone section (Table 15), no significant interaction effects were found across all parameters (*p* > 0.05). However, significant main effects of energy density were observed for several cortical bone parameters. Hens fed standard-energy diets showed higher BMD (*p* = 0.035) and BV/TV (*p* = 0.044) compared to those on low-energy diets. However, low-energy diets were associated with increased volume of open pores (*p* = 0.017), open porosity (*p* = 0.036), volume of total pores (*p* = 0.018), and total porosity (*p* = 0.044). No significant main effects of corn type were observed (*p* > 0.05).

For the trabecular bone section (Table 16), no significant interaction effects or main effects of energy density or corn type were observed for BMD, BMC, BV, trabecular thickness, trabecular number, trabecular pattern factor, connectivity density, degree of anisotropy, or structure model index (*p* > 0.05).

At 45 weeks of age (Table 17), the microarchitecture of the femoral medullary section was not significantly affected by dietary energy density, corn type, or their interaction (*p* > 0.05).

## 4. Discussion

This experiment compared two ME densities (standard vs. 200 kcal/kg lower) and two corn types (conventional vs. Enogen corn) in laying hens across 18–45 weeks of age; birds received the same treatment diets from 0 to 18 weeks, and those results were reported previously [25]. The Enogen corn used in this study is derived from corn event 3272, which was developed to express a thermostable AMY797E α-amylase mainly in the kernel endosperm rather than in pollen and leaves [41]. Regulatory assessments describe AMY797E α-amylase as an enzyme that hydrolyzes starch by cleaving internal α-1,4-glucosidic bonds, producing smaller carbohydrates such as dextrin, maltose, and glucose [42]. Furthermore, the compositional comparisons of event 3272 grain (proximate, amino acids, fatty acids, minerals, vitamins, and anti-nutrients/secondary metabolites) concluded that this corn grain is compositionally equivalent to non-modified and commercial corn, supporting the expectation that any biological response in poultry would mainly arise from improved digestion and availability effects rather than large intrinsic nutrient-composition changes [43]. In laying hens, dietary energy density is a major contributing factor of voluntary feed intake, and hens often attempt to adjust feed intake to maintain energy intake when ME changes, although the compensation can be incomplete and strain dependent [44]. Because dietary fat sources, such as soybean oil, are highly energy-dense and are used to increase dietary energy and influence digesta kinetics and fat-soluble nutrient absorption, replacing oil with inert diluent is expected to lower dietary energy density and may increase feed intake and worsen feed conversion when energy compensation is incomplete [45].

A major finding of the current study was that hens fed Enogen corn consistently showed better feed efficiency than those fed conventional corn. Across the laying cycle, Enogen-based diets resulted in lower FCR per dozen eggs, with benefits observed from 26 weeks of age onwards, which indicates that the thermostable α-amylase (AMY797E) trait in expressed in Enogen corn could have improved starch utilization [46], allowing hens to produce more eggs with less feed. Although direct layer trials with amylase-expressing corn are limited in the literature, poultry studies with Enogen feed corn in broiler settings indicate that corn type can interact with feed-manufacturing conditions and starch properties, providing a digestion pathway by which amylase-expressing corn could alter effective energy utilization and feed efficiency [21]. Furthermore, the improvement in FCR aligns with studies on exogenous enzymes supplementation, which reported that exogenous enzymes can increase nutrient digestibility and performance by improving breakdown of dietary substrates that are not fully utilized by endogenous enzymes alone efficiently [47]. The α-amylase enzyme can hydrolyzes α-1,4 glycosidic bonds in starch, yielding maltose, maltotriose and α-dextrin, which are then further hydrolyzed by brush border enzymes into glucose [48,49] and readily absorbed in the small intestine, increasing the effective energy value of the diet [50]. The response observed in the current laying hens differs from previous broiler studies in which Enogen corn did not improve FCR but improved the body weight and feed intake [21]. The differential response of broiler and laying hens could be species and diet dependent. Broilers are slaughtered at 5–7 weeks of age, during which endogenous amylase secretion is already high [51], and much of the additional glucose liberated from starch is utilized for rapid muscle accretion and fat deposition rather than improving efficiency [52]. However, laying hens regulate feed intake tightly to meet daily energy requirements for egg production [53]. Thus, any increase in starch digestibility from Enogen corn directly improves the efficiency of egg production. Moreover, endogenous amylase activity tends to decline with age in layers [54], making the effects of amylase more apparent during mid to late-lay.

Body weight and body weight gain remained unaffected across treatments from 18–45 weeks, which supports the ability of laying hens to regulate feed intake and maintain growth [44]. Commercial White Leghorn hens are genetically selected for high egg production rather than growth, and by sexual maturity, their growth curve plateaus, and further weight gain is minimal [55]. Thus, even dietary modifications have limited effect on body weight once birds enter egg production age. In the current study, hens on low-energy diets consumed more feed, particularly during 36–45 weeks of age, which indicates a compensatory feed intake response to maintain energy balance. As previously discussed, in laying hens, feed intake regulation is a well-recognized response to dietary energy level. When hens are offered reduced-energy diets, they typically increase feed intake to compensate and maintain daily ME intake [56,57]. Despite this increased intake, hens on low-ME diets resulted in a higher FCR than those on standard-ME diets, indicating that compensation was incomplete and efficiency was compromised when energy density was lowered.

In addition to improved feed efficiency, Enogen corn also supported higher HDEP between 35 and 45 weeks of age compared with conventional corn. Enogen-fed hens maintained laying rates 8–10 percentage points higher than controls, and by 45 weeks, they produced heavier eggs with greater yolk weight. These results align with a previous study showing improved egg production when diets are supplemented with carbohydrase enzyme [58], which suggests that Enogen corn functions in a similar manner as an intrinsic carbohydrase enzyme that improves starch digestion and provides more available energy for egg formation. The observed improvements also indicate greater ME availability with Enogen corn, supporting the energy-intensive processes of follicular growth, ovulation, and yolk deposition in laying hens [59,60]. However, eggshell thickness and specific gravity were unaffected by the corn type and energy density. This could be because eggshell quality is mainly affected by dietary mineral intake rather than moderate energy adjustments [61,62]. Also, reducing dietary energy by 200 kcal/kg had no significant effect on egg production, as hens compensated with higher feed intake to maintain daily energy supply.

Body composition of laying hens was not affected by the treatments. Similar to the body weight changes, no significant effects of energy density or corn type were observed on body composition at 45 weeks. The lack of fat or muscle differences suggests that increased ME from Enogen corn was directed toward egg production rather than storage in body reserves [60]. Similarly, hens on low-energy diets compensated by increasing feed intake, potentially preserving their body composition [56].

However, in the femur bone, low-energy diets decreased BMD, bone volume fraction and increased porosity in the cortical region, which indicate that low-energy diet has potential to compromise bone integrity. Low-energy diets can negatively affect bone quality because hens must prioritize available energy and nutrients for egg production, often at the expense of bone maintenance. Cortical bone mineralization is an energy-intensive process requiring adequate dietary energy to support intestinal calcium absorption, active ion transport, and bone matrix formation [63,64]. When dietary energy is reduced, hens may increase feed intake, but compensation is often incomplete, resulting in lower efficiency of nutrient utilization [56,65]. This limited energy supply can impair osteoblast activity, leading to reduced BMD and bone volume fraction [66].

From a broader perspective, the results of current study show the potential of Enogen corn as a nutritional strategy to improve egg production efficiency in laying hens. On the other hand, a moderate reduction in dietary energy (200 kcal/kg) was generally tolerated in laying hens in terms of egg production metrices and body weight responses, providing producers with flexibility to lower feed costs when energy-rich ingredients are expensive. However, cost analyses should be conducted to determine whether ingredient savings outweigh the additional feed intake.

Future studies should investigate a wider range of dietary energy reductions. The 200 kcal/kg reduction used in the current study was well tolerated in terms of egg production metrices and body weight responses, but more severe deficits of 300 kcal/kg or more and stress conditions such as heat stress, mild coccidiosis might show stronger performance benefits from Enogen corn. Similarly, extending the experimental period beyond 45 weeks would clarify whether Enogen-fed hens can sustain egg production and maintain bone health through late laying cycles.

## 5. Conclusions

In conclusion, the current study shows that replacing conventional corn with thermostable α-amylase corn (AMY797E) improved feed efficiency across the laying period and supported higher hen-day egg production, with heavier eggs and greater yolk weight. These performance gains occurred without altering body weight and whole-body composition. A 200 kcal/kg reduction in dietary ME was largely tolerated through compensatory feed intake, but it consistently worsened FCR and compromised the cortical bone quality.

## Figures and Tables

**Table 1:** Analyzed composition of conventional and Enogen corn (as-fed basis).

Component	Conventional Corn ^1^	Enogen Corn ^2^
Gross energy (kcal/kg)	3861	3935
TMEn (kcal/kg)	3394	3483
CP (%)	7.45	7.78
Digestible amino acids (%)		
Alanine	0.42	0.43
Arginine	0.28	0.27
Aspartic acid	0.37	0.38
Cysteine	0.12	0.11
Glutamic acid	1.13	1.14
Glycine	0.02	0.15
Histidine	0.18	0.18
Isoleucine	0.19	0.21
Leucine	0.70	0.74
Lysine	0.18	0.20
Methionine	0.15	0.12
Phenylalanine	0.28	0.30
Proline	0.53	0.56
Serine	0.27	0.27
Threonine	0.19	0.20
Tryptophan	0.04	0.04
Tyrosine	0.17	0.18
Valine	0.24	0.26

^1^ Conventional corn: standard corn without genetic modification. ^2^ Enogen corn: genetically modified corn incorporated with thermostable α-amylase enzyme (AMY797E) directly into the corn endosperm. Abbreviations: TMEn, true metabolizable energy (nitrogen-corrected); CP, crude protein.

**Table 2:** Ingredients and calculated nutrient composition of peaking phase diets (as-fed basis).

Ingredient (%)	Standard-Energy Diets ^1^		Low-Energy Diets ^2^	
	Conventional ^3^	Enogen ^4^	Conventional	Enogen
Enogen corn	0.00	56.18	0.00	56.18
Conventional corn	56.18	0.00	56.18	0.00
Soybean meal, 48% CP	26.11	26.11	26.11	26.11
Soybean oil	5.37	5.37	3.09	3.09
Sand	0.00	0.00	2.28	2.27
Limestone ^5^	10.43	10.43	10.43	10.43
Defluorinated phosphate	0.79	0.79	0.79	0.79
Salt	0.30	0.30	0.30	0.30
DL-Methionine	0.19	0.20	0.19	0.21
L-Lysine HCl	0.03	0.02	0.03	0.02
L-Threonine	0.02	0.02	0.02	0.02
Mineral premix ^6^	0.08	0.08	0.08	0.08
Vitamin premix ^7^	0.50	0.50	0.50	0.50
Total	100.00	100.00	100.00	100.00
Calculated composition				
ME (kcal/kg)	3000	3080	2800	2880
CP (%)	17.76	17.50	17.76	17.50
Crude fiber (%)	2.25	2.25	2.25	2.25
Calcium (%)	4.30	4.30	4.30	4.30
Available phosphorus (%)	0.47	0.47	0.47	0.47
Digestible amino acids (%)				
Methionine	0.44	0.44	0.44	0.44
TSAA	0.75	0.77	0.75	0.77
Lysine	0.84	0.84	0.84	0.84
Threonine	0.59	0.59	0.59	0.59
Tryptophan	0.21	0.21	0.21	0.21
Valine	0.78	0.78	0.78	0.78
Arginine	1.02	1.02	1.02	1.02

^1^ Standard-energy diets: diets formulated to meet or exceed metabolizable energy (ME) requirement during peaking phase (18–37 weeks) of White Leghorn hens according to breeder guideline. ^2^ Low-energy diets: diets formulated to reduce metabolizable energy (ME) by 200 kcal/kg compared to standard-energy diets. This reduction was achieved by decreasing the soybean oil content and replacing it with inert sand as a filler. ^3^ Conventional: diet formulated with conventional corn as the primary carbohydrate source. ^4^ Enogen: diet formulated with genetically modified Enogen corn with thermostable α-amylase enzyme (AMY797E) directly incorporated into the corn endosperm as the primary carbohydrate source. ^5^ Limestone: 50% fine limestone, 50% coarse oystershell. ^6^ Mineral premix (per kg diet): Mn (107.2 mg), Zn (85.6 mg), Fe (21.04 mg), Cu (3.2 mg), I (0.8 mg), Se (0.32 mg). ^7^ Vitamin premix (per kg diet): Vitamin A (17,637 IU), D_3_ (7000 ICU), E (14.9 IU), B_12_ (0.044 mg), Menadione (5.51 mg), Riboflavin (17.6 mg), d-Pantothenic Acid (27.3 mg), Thiamine (4.85 mg), Niacin (101.4 mg), B_6_ (7.3 mg), Folic Acid (2.86 mg), and Biotin (0.395 mg). Abbreviations: ME, metabolizable energy; CP, crude protein; TSAA, total sulfur amino acids.

**Table 3:** Ingredients and calculated nutrient composition of layer 2 phase diets (as-fed basis).

Ingredient (%)	Standard-Energy Diets ^1^		Low-Energy Diets ^2^	
	Conventional ^3^	Enogen ^4^	Conventional	Enogen
Enogen corn	0.00	53.58	0.00	53.58
Conventional corn	53.58	0.00	53.58	0.00
Soybean meal, 48% CP	22.42	22.42	22.42	22.42
Soybean oil	6.00	6.00	3.73	3.73
Sand	5.74	5.74	8.01	8.01
Limestone ^5^	10.30	10.30	10.30	10.30
Defluorinated phosphate	0.82	0.82	0.82	0.82
Salt	0.30	0.30	0.30	0.30
DL-Methionine	0.19	0.19	0.19	0.19
L-Lysine HCl	0.03	0.03	0.03	0.03
L-Threonine	0.04	0.04	0.04	0.04
Mineral premix ^6^	0.08	0.08	0.08	0.08
Vitamin premix ^7^	0.50	0.50	0.50	0.50
Total	100.00	100.00	100.00	100.00
Calculated composition				
ME, kcal/kg	2900	2990	2700	2790
CP (%)	16.30	16.30	16.30	16.40
Crude fiber (%)	2.05	2.05	2.05	2.05
Calcium (%)	4.25	4.25	4.25	4.25
Available phosphorus (%)	0.47	0.47	0.47	0.47
Digestible amino acids (%)				
Methionine	0.41	0.39	0.41	0.39
TSAA	0.69	0.69	0.69	0.69
Lysine	0.77	0.77	0.77	0.77
Threonine	0.54	0.54	0.54	0.54
Tryptophan	0.18	0.18	0.18	0.18
Valine	0.68	0.69	0.68	0.69
Arginine	0.89	0.89	0.89	0.89

^1^ Standard-energy diets: diets formulated to meet or exceed metabolizable energy (ME) requirement during layer 2 phase (38–45 weeks) of White Leghorn hens according to breeder guideline. ^2^ Low-energy diets: Diets formulated to reduce metabolizable energy (ME) by 200 kcal/kg compared to standard-energy diets. This reduction was achieved by decreasing the soybean oil content and replacing it with inert sand as a filler. ^3^ Conventional: diet formulated with conventional corn as the primary carbohydrate source. ^4^ Enogen: diet formulated with genetically modified Enogen corn with thermostable α-amylase enzyme (AMY797E) directly incorporated into the corn endosperm as the primary carbohydrate source. ^5^ Limestone: 40% fine limestone, 60% coarse oystershell. ^6^ Mineral premix (per kg diet): Mn (107.2 mg), Zn (85.6 mg), Fe (21.04 mg), Cu (3.2 mg), I (0.8 mg), Se (0.32 mg). ^7^ Vitamin premix (per kg diet): Vitamin A (17,637 IU), D_3_ (7000 ICU), E (14.9 IU), B_12_ (0.044 mg), Menadione (5.51 mg), Riboflavin (17.6 mg), d-Pantothenic Acid (27.3 mg), Thiamine (4.85 mg), Niacin (101.4 mg), B_6_ (7.3 mg), Folic Acid (2.86 mg), and Biotin (0.395 mg). Abbreviations: ME, metabolizable energy; CP, crude protein; TSAA, total sulfur amino acids.

**Table 4:** Body weight of laying hens fed conventional corn or Enogen corn diets with standard or reduced energy.

Parameters		Body Weight (kg)			
		Week 18	Week 25	Week 35	Week 45
***Interaction effect***					
**Energy density ^1^**	**Corn type ^2^**				
Standard	Conventional	1.276	1.547	1.589	1.632
	Enogen	1.280	1.547	1.568	1.611
Low	Conventional	1.261	1.521	1.573	1.611
	Enogen	1.287	1.543	1.600	1.635
SEM		0.011	0.016	0.025	0.026
*p* (Interaction)		0.302	0.487	0.343	0.403
***Main effects***					
**Energy density**					
Standard		1.278	1.547	1.578	1.621
Low		1.274	1.532	1.586	1.623
**Corn type**					
Conventional		1.269	1.534	1.581	1.622
Enogen		1.284	1.545	1.584	1.623
SEM		0.008	0.012	0.017	0.019
*p* (Energy density)		0.722	0.371	0.902	0.944
*p* (Corn type)		0.172	0.504	0.343	0.966

^1^ Energy density: diets were formulated at two metabolizable energy (ME) levels: standard (meeting or exceeding breeder recommendations for White Leghorn hens) and low (200 kcal/kg lower than standard, achieved by substituting soybean oil with inert sand). ^2^ Corn type: diets were based on either conventional corn or Enogen corn, a genetically modified variety expressing a thermostable α-amylase (AMY797E) directly in the endosperm. No significant main or interaction effects of energy density or corn type on body weight were observed at 25, 35, or 45 weeks of age (*p* > 0.05; n = 10).

**Table 5:** Body weight gain of laying hens fed conventional corn or Enogen corn diets with standard or reduced energy.

Parameters		Body Weight Gain (kg)			
		Week 18–25	Week 26–35	Week 36–45	Week 18–45
***Interaction effect***					
**Energy density ^1^**	**Corn type ^2^**				
Standard	Conventional	0.271	0.041	0.043	0.355
	Enogen	0.267	0.021	0.043	0.331
Low ^1^	Conventional	0.260	0.052	0.038	0.350
	Enogen	0.256	0.056	0.035	0.347
SEM		0.011	0.016	0.011	0.024
*p* (Interaction)		0.987	0.449	0.899	0.643
***Main effects***					
**Energy density**					
Standard		0.269	0.031	0.043	0.343
Low		0.258	0.054	0.037	0.349
**Corn type**					
Conventional		0.265	0.047	0.041	0.353
Enogen		0.261	0.039	0.039	0.339
SEM		0.008	0.011	0.008	0.017
*p* (Energy density)		0.342	0.163	0.557	0.813
*p* (Corn type)		0.726	0.627	0.860	0.567

^1^ Energy density: diets were formulated at two metabolizable energy (**ME**) levels: standard (meeting or exceeding breeder recommendations for White Leghorn hens) and low (200 kcal/kg lower than standard, achieved by substituting soybean oil with inert sand). ^2^ Corn type: diets were based on either conventional corn or Enogen corn, a genetically modified variety expressing a thermostable α-amylase (AMY797E) directly in the endosperm. No significant main or interaction effects of energy density or corn type on body weight gain were observed at 25, 35, or 45 weeks of age (*p* > 0.05; n = 10).

**Table 6:** Feed intake of laying hens fed conventional corn or Enogen corn diets with standard or reduced energy.

Parameters		Feed Intake (kg)			
		Week 18–25	Week 26–35	Week 36–45	Week 18–45
***Interaction effect***					
**Energy density ^1^**	**Corn type ^2^**				
Standard	Conventional	4.445	7.792	8.864 ^ab^	21.101
	Enogen	4.393	7.732	8.687 ^b^	20.812
Low	Conventional	4.458	7.732	8.882 ^ab^	21.073
	Enogen	4.344	7.632	9.023 ^a^	20.998
SEM		0.089	0.109	0.063	0.198
*p* (Interaction)		0.726	0.853	0.017	0.593
***Main effects***					
**Energy density**					
Standard		4.419	7.762	8.776 ^b^	20.957
Low		4.401	7.682	8.952 ^a^	21.035
**Corn type**					
Conventional		4.452	7.762	8.873	21.087
Enogen		4.368	7.682	8.855	20.905
SEM		0.063	0.077	0.045	0.140
*p* (Energy density)		0.841	0.469	0.008	0.694
*p* (Corn type)		0.354	0.469	0.777	0.366

^1^ Energy density: diets were formulated at two metabolizable energy (**ME**) levels: standard (meeting or exceeding breeder recommendations for White Leghorn hens) and low (200 kcal/kg lower than standard, achieved by substituting soybean oil with inert sand). ^2^ Corn type: diets were based on either conventional corn or Enogen corn, a genetically modified variety expressing a thermostable α-amylase (AMY797E) directly in the endosperm. ^a,b^ Values within a column with different superscripts differ significantly (*p* < 0.05; n = 10). Significant interactions of energy density and corn type were observed on feed intake during 36–45 weeks of age (*p* < 0.05).

**Table 7:** Feed conversion ratio of laying hens fed conventional corn or Enogen corn diets with standard or reduced energy.

Parameters		FCR/Dozen Eggs			
		Week 18–25	Week 26–35	Week 36–45	Week 18–45
***Interaction effect***					
**Energy density ^1^**	**Corn type ^2^**				
Standard	Conventional	2.38	3.70	3.58	3.22
	Enogen	2.36	3.15	3.31	2.94
Low	Conventional	2.38	3.91	3.83	3.37
	Enogen	2.37	3.39	3.58	3.12
SEM		0.05	0.14	0.08	0.07
*p* (Interaction)		0.949	0.901	0.904	0.885
***Main effects***					
**Energy density**					
Standard		2.37	3.43	3.44 ^b^	3.08 ^b^
Low		2.38	3.65	3.71 ^a^	3.24 ^a^
**Corn type**					
Conventional		2.38	3.81 ^a^	3.70 ^a^	3.30 ^a^
Enogen		2.37	3.27 ^b^	3.44 ^b^	3.03 ^b^
SEM		0.04	0.10	0.06	0.05
*p* (Energy density)		0.917	0.128	0.004	0.030
*p* (Corn type)		0.846	<0.001	0.004	<0.001

^1^ Energy density: diets were formulated at two metabolizable energy (**ME**) levels: standard (meeting or exceeding breeder recommendations for White Leghorn hens) and low (200 kcal/kg lower than standard, achieved by substituting soybean oil with inert sand). ^2^ Corn type: diets were based on either conventional corn or Enogen corn, a genetically modified variety expressing a thermostable α-amylase (AMY797E) directly in the endosperm. ^a,b^ Values within a column with different superscripts differ significantly (*p* < 0.05; n = 10). Significant main effects of corn type were observed on feed conversion ratio (FCR) per dozen eggs during weeks 26–35, 36–45, and 18–45 of age, whereas significant main effects of energy density were observed during weeks 36–45 and 18–45 of age (*p* < 0.05).

**Table 8:** Egg quality parameters of laying hens fed conventional corn or Enogen corn diets with standard or reduced energy at 25 weeks of age.

Parameters		Week 25										
		EW (g)	YW (g)	ESW (g)	AW (g)	SG (g/cm^3^)	EST (mm)	Y%	ES %	A%	HDEP (%)	HU
***Interaction effect***												
**Energy density ^1^**	**Corn type ^2^**											
Standard	Conventional	54.4	13.7	5.7	35.0	1.092	0.398	25.2	10.5	64.3	90.6	95.6
	Enogen	55.2	13.6	5.8	35.8	1.093	0.401	24.7	10.4	64.9	90.0	95.1
Low	Conventional	55.4	13.6	5.9	35.9	1.093	0.408	24.5	10.7	64.8	90.6	98.4
	Enogen	54.2	13.6	5.6	35.0	1.093	0.398	25.0	10.4	64.6	90.0	95.4
SEM		0.6	0.2	0.1	0.5	0.001	0.005	0.3	0.2	0.4	1.4	0.8
*p* (Interaction)		0.870	0.870	0.189	0.067	0.145	0.266	0.137	0.626	0.289	0.977	0.146
***Main effects***												
**Energy density**												
Standard		54.8	13.7	5.8	35.4	1.093	0.400	24.9	10.5	64.6	90.3	95.4
Low		54.8	13.6	5.8	35.5	1.093	0.403	24.8	10.5	64.7	90.3	96.9
**Corn type**												
Conventional		54.9	13.6	5.8	35.5	1.093	0.403	24.9	10.6	64.6	90.6	97.0 ^a^
Enogen		54.7	13.6	5.7	35.4	1.093	0.400	24.9	10.4	64.7	90.0	95.3 ^b^
SEM		0.4	0.1	0.1	0.3	0.0004	0.004	0.2	0.1	0.3	1.0	0.6
*p* (Energy density)		0.682	0.682	0.883	0.875	0.725	0.506	0.656	0.899	0.747	0.996	0.068
*p* (Corn type)		0.806	0.806	0.305	0.930	0.623	0.506	0.971	0.304	0.661	0.654	0.040

^1^ Energy density: diets were formulated at two metabolizable energy (**ME**) levels: standard (meeting or exceeding breeder recommendations for White Leghorn hens) and low (200 kcal/kg lower than standard, achieved by substituting soybean oil with inert sand). ^2^ Corn type: diets were based on either conventional corn or Enogen corn, a genetically modified variety expressing a thermostable α-amylase (AMY797E) directly in the endosperm. ^a,b^ Values within a column with different superscripts differ significantly (*p* < 0.05; n = 30). Significant main effects of corn type were observed on Haugh unit during 25 weeks of age (*p* < 0.05). Abbreviations: EW = egg weight; YW = yolk weight; ESW = eggshell weight; AW = albumen weight; SG = specific gravity; EST = eggshell thickness; Y% = yolk percentage; ES% = eggshell percentage; A% = albumen percentage; HDEP = hen-day egg production; HU = Haugh unit.

**Table 9:** Egg quality parameters of laying hens fed conventional corn or Enogen corn diets with standard or reduced energy at 30 weeks of age.

Parameters		Week 30										
		EW (g)	YW (g)	ESW (g)	AW (g)	SG (g/cm^3^)	EST (mm)	Y%	ES%	A%	HDEP (%)	HU
***Interaction effect***												
**Energy density ^1^**	**Corn type ^2^**											
Standard	Conventional	55.8	14.5	5.8	35.5	1.090	0.400	26.0	10.5	63.5	96.7	83.0
	Enogen	54.8	14.2	5.9	34.7	1.090	0.402	26.0	10.7	63.3	96.3	82.7
Low	Conventional	56.6	15.5	6.0	35.1	1.088	0.409	27.5	10.6	61.9	96.0	79.9
	Enogen	55.5	14.3	5.7	35.5	1.089	0.400	25.8	10.4	63.8	96.5	82.9
SEM		1.1	0.4	0.1	1.2	0.002	0.005	0.8	0.3	1.0	0.5	2.0
*p* (Interaction)		0.965	0.210	0.189	0.641	0.780	0.256	0.295	0.452	0.280	0.396	0.413
***Main effects***												
**Energy density**												
Standard		55.3	14.4	5.8	35.1	1.090	0.401	26.0	10.6	63.4	96.5	82.8
Low		56.1	14.9	5.9	35.3	1.088	0.404	26.7	10.5	62.8	96.2	81.4
**Corn type**												
Conventional		56.2	15.0 ^a^	5.9	35.3	1.089	0.405	26.8	10.5	62.7	96.3	81.4
Enogen		55.2	14.3 ^b^	5.8	35.1	1.089	0.401	25.9	10.6	63.5	96.4	82.8
SEM		0.8	0.2	0.1	0.8	0.001	0.004	0.5	0.2	0.7	0.3	
*p* (Energy density)		0.514	0.127	0.883	0.876	0.404	0.531	0.404	0.781	0.569	0.620	0.489
*p* (Corn type)		0.362	0.040	0.305	0.876	1.000	0.488	0.293	0.968	0.411	0.824	0.499

^1^ Energy density: diets were formulated at two metabolizable energy (**ME**) levels: standard (meeting or exceeding breeder recommendations for White Leghorn hens) and low (200 kcal/kg lower than standard, achieved by substituting soybean oil with inert sand). ^2^ Corn type: diets were based on either conventional corn or Enogen corn, a genetically modified variety expressing a thermostable α-amylase (AMY797E) directly in the endosperm. ^a,b^ Values within a column with different superscripts differ significantly (*p* < 0.05; n = 30). Significant main effects of corn type were observed on yolk weight during 30 weeks of age (*p* < 0.05). Abbreviations: EW = egg weight; YW = yolk weight; ESW = eggshell weight; AW = albumen weight; SG = specific gravity; EST = eggshell thickness; Y% = yolk percentage; ES% = eggshell percentage; A% = albumen percentage; HDEP = hen-day egg production; HU = Haugh unit.

**Table 10:** Egg quality parameters of laying hens fed conventional corn or Enogen corn diets with standard or reduced energy at 35 weeks of age.

Parameters		Week 35										
		EW (g)	YW (g)	ESW (g)	AW (g)	SG (g/cm^3^)	EST (mm)	Y%	ES%	A%	HDEP (%)	HU
***Interaction effect***												
**Energy density ^1^**	**Corn type ^2^**											
Standard	Conventional	57.7	15.5	5.9	36.3	1.083	0.402	26.8	10.3	62.9	81.2	90.9
	Enogen	59.4	16.2	6.0	37.2	1.087	0.404	27.3	10.0	62.7	93.3	90.0
Low	Conventional	58.2	14.9	6.1	37.2	1.086	0.411	25.7	10.5	63.8	77.6	90.0
	Enogen	58.8	16.2	5.8	36.8	1.087	0.402	27.5	9.9	62.5	86.0	87.3
SEM		0.9	0.4	0.1	0.8	0.001	0.005	0.6	0.3	0.7	2.9	1.8
*p* (Interaction)		0.541	0.438	0.189	0.395	0.460	0.256	0.230	0.520	0.429	0.520	0.608
***Main effects***												
**Energy density**												
Standard		58.6	15.9	5.9	36.8	1.085	0.403	27.1	10.2	62.8	87.2	90.5
Low		58.5	15.6	6.0	37.0	1.086	0.406	26.6	10.2	63.2	81.8	88.6
**Corn type**												
Conventional		58.0	15.2 ^b^	6.0	36.7	1.084	0.407	26.3	10.4	63.3	79.4 ^b^	90.5
Enogen		59.1	16.2 ^a^	5.9	37.0	1.087	0.403	27.4	10.0	62.6	89.6 ^a^	88.6
SEM		0.6	0.3	0.1	0.6	0.001	0.004	0.4	0.2	0.5	2.1	1.3
*p* (Energy density)		0.956	0.438	0.883	0.776	0.270	0.531	0.448	0.834	0.567	0.070	0.315
*p* (Corn type)		0.205	0.013	0.305	0.745	0.070	0.488	0.057	0.124	0.285	0.001	0.306

^1^ Energy density: diets were formulated at two metabolizable energy (**ME**) levels: standard (meeting or exceeding breeder recommendations for White Leghorn hens) and low (200 kcal/kg lower than standard, achieved by substituting soybean oil with inert sand). ^2^ Corn type: diets were based on either conventional corn or Enogen corn, a genetically modified variety expressing a thermostable α-amylase (AMY797E) directly in the endosperm. ^a,b^ Values within a column with different superscripts differ significantly (*p* < 0.05; n = 30). Significant main effects of corn type were observed on yolk weight and hen-day egg production during 35 weeks of age (*p* < 0.05). Abbreviations: EW = egg weight; YW = yolk weight; ESW = eggshell weight; AW = albumen weight; SG = specific gravity; EST = eggshell thickness; Y% = yolk percentage; ES% = eggshell percentage; A% = albumen percentage; HDEP = hen-day egg production; HU = Haugh unit.

**Table 11:** Egg quality parameters of laying hens fed conventional corn or Enogen corn diets with standard or reduced energy at 40 weeks of age.

Parameters		Week 40										
		EW (g)	YW (g)	ESW (g)	AW (g)	SG (g/cm^3^)	EST (mm)	Y%	ES%	A%	HDEP (%)	HU
***Interaction effect***												
**Energy density ^1^**	**Corn type ^2^**											
Standard	Conventional	60.4	16.2	6.0	38.2	1.084	0.393	26.8	10.0	63.2	84.1	83.2
	Enogen	57.7	16.2	5.7	35.8	1.084	0.389	28.1	9.9	62.1	92.5	83.2
Low	Conventional	60.1	16.3	5.8	37.7	1.085	0.380	27.3	9.7	63.0	78.9	81.8
	Enogen	59.7	17.0	5.6	37.1	1.086	0.388	28.5	9.4	62.1	87.5	82.5
SEM		1.1	0.5	0.2	0.8	0.002	0.008	0.6	0.3	0.5	2.6	1.5
*p* (Interaction)		0.322	0.477	0.767	0.286	0.882	0.442	0.974	0.700	0.796	0.947	0.806
***Main effects***												
**Energy density**												
Standard		59.1	16.2	5.9	37.0	1.084	0.391	27.4	9.9	62.6	88.3	83.2
Low		59.9	16.7	5.7	37.4	1.085	0.384	27.9	9.5	62.6	83.2	82.1
**Corn type**												
Conventional		60.3	16.3	5.9	37.9	1.085	0.387	27.1 ^b^	9.8	63.1	81.5 ^b^	82.5
Enogen		58.7	16.6	5.7	36.5	1.085	0.389	28.3 ^a^	9.6	62.1	90.0 ^a^	82.8
SEM		0.8	0.3	0.1	0.6	0.001	0.006	0.4	0.2	0.4	1.8	1.0
*p* (Energy density)		0.463	0.321	0.377	0.654	0.459	0.371	0.422	0.175	0.868	0.055	0.473
*p* (Corn type)		0.184	0.477	0.144	0.089	0.882	0.797	0.043	0.503	0.062	0.002	0.820

^1^ Energy density: diets were formulated at two metabolizable energy (**ME**) levels: standard (meeting or exceeding breeder recommendations for White Leghorn hens) and low (200 kcal/kg lower than standard, achieved by substituting soybean oil with inert sand). ^2^ Corn type: diets were based on either conventional corn or Enogen corn, a genetically modified variety expressing a thermostable α-amylase (AMY797E) directly in the endosperm. ^a,b^ Values within a column with different superscripts differ significantly (*p* < 0.05; n = 30). Significant main effects of corn type were observed on yolk percentage and hen-day egg production during 40 weeks of age (*p* < 0.05). Abbreviations: EW = egg weight; YW = yolk weight; ESW = eggshell weight; AW = albumen weight; SG = specific gravity; EST = eggshell thickness; Y% = yolk percentage; ES% = eggshell percentage; A% = albumen percentage; HDEP = hen-day egg production; HU = Haugh unit.

**Table 12:** Egg quality parameters of laying hens fed conventional corn or Enogen corn diets with standard or reduced energy at 45 weeks of age.

Parameters		Week 45										
		EW (g)	YW (g)	ESW (g)	AW (g)	SG (g/cm^3^)	EST (mm)	Y%	ES%	A%	HDEP (%)	HU
***Interaction effect***												
**Energy density ^1^**	**Corn type ^2^**											
Standard	Conventional	55.8	17.0	5.9	33.8 ^b^	1.067	0.382	29.9 ^ab^	10.5	59.3 ^ab^	85.6	93.7
	Enogen	61.3	17.6	6.2	36.9 ^a^	1.071	0.389	29.1 ^ab^	10.1	60.8 ^a^	90.3	94.4
Low	Conventional	57.7	16.4	5.8	35.3 ^ab^	1.070	0.390	28.6 ^b^	10.1	61.3 ^a^	80.0	93.8
	Enogen	58.9	18.0	6.2	33.8 ^b^	1.071	0.398	31.0 ^a^	10.6	58.3 ^b^	86.9	93.8
SEM		1.1	0.5	0.2	0.9	0.002	0.010	0.7	0.3	0.8	2.3	1.9
*p* (Interaction)		0.054	0.325	0.743	0.018	0.440	0.969	0.036	0.063	0.010	0.645	0.842
***Main effects***												
**Energy density**												
Standard		58.6	17.3	6.1	35.3	1.069	0.386	29.5	10.3	60.1	88.0	94.0
Low		58.3	17.2	6.0	34.6	1.071	0.394	29.8	10.3	59.8	83.4	93.8
**Corn type**												
Conventional		56.8 ^b^	16.7 ^b^	5.9	34.5	1.068	0.386	29.3	10.3	60.3	82.8 ^b^	93.8
Enogen		60.1 ^a^	17.8 ^a^	6.2	35.4	1.071	0.393	30.1	10.3	59.6	88.6 ^a^	94.1
SEM		0.8	0.3	0.1	0.6	0.001	0.007	0.5	0.2	0.6	1.6	1.3
*p* (Energy density)		0.815	0.847	0.835	0.435	0.274	0.399	0.674	0.988	0.770	0.053	0.889
*p* (Corn type)		0.004	0.026	0.059	0.385	0.176	0.457	0.284	0.878	0.358	0.015	0.869

^1^ Energy density: diets were formulated at two metabolizable energy (**ME**) levels: standard (meeting or exceeding breeder recommendations for White Leghorn hens) and low (200 kcal/kg lower than standard, achieved by substituting soybean oil with inert sand). ^2^ Corn type: diets were based on either conventional corn or Enogen corn, a genetically modified variety expressing a thermostable α-amylase (AMY797E) directly in the endosperm. ^a,b^ Values within a column with different superscripts differ significantly (*p* < 0.05; n = 30). Significant interaction effects of energy density and corn type were observed on albumen weight, yolk percentage and albumen percentage, whereas main effects of corn type were observed on egg weight, yolk weight and hen-day egg production during 45 weeks of age (*p* < 0.05). Abbreviations: EW = egg weight; YW = yolk weight; ESW = eggshell weight; AW = albumen weight; SG = specific gravity; EST = eggshell thickness; Y% = yolk percentage; ES% = eggshell percentage; A% = albumen percentage; HDEP = hen-day egg production; HU = Haugh unit.

**Table 13:** Body composition of laying hens fed conventional corn or Enogen corn diets with standard or reduced energy at 45 weeks of age.

Parameters		BMD (g/cm^2^)	BMC (g)	Bone Area (cm^2^)	Fat (%)	Fat (kg)	Muscle (%)	Muscle (kg)	Fat + Muscle (kg)
***Interaction effect***									
**Energy density ^1^**	**Corn type ^2^**								
Standard	Conventional	0.234	47.9	184.5	47.9	0.702	52.1	0.755	1.457
	Enogen	0.234	47.4	181.4	48.4	0.729	51.6	0.767	1.496
Low	Conventional	0.236	47.7	181.1	47.2	0.710	52.8	0.784	1.494
	Enogen	0.236	48.7	187.0	48.5	0.738	51.5	0.778	1.516
SEM		0.005	1.4	3.5	1.6	0.044	1.6	0.026	0.056
*p* (Interaction)		0.991	0.613	0.210	0.826	0.987	0.825	0.733	0.885
***Main effects***									
**Energy density**									
Standard		0.234	47.6	183.0	48.1	0.715	51.8	0.761	1.476
Low		0.236	48.2	184.1	47.8	0.724	52.2	0.781	1.505
**Corn type**									
Conventional		0.235	47.8	182.8	47.5	0.706	52.5	0.769	1.475
Enogen		0.235	48.1	184.2	48.5	0.734	51.5	0.772	1.506
SEM		0.003	1.0	2.5	1.1	0.031	1.1	0.018	0.040
*p* (Energy density)		0.647	0.693	0.757	0.843	0.841	0.842	0.457	0.616
*p* (Corn type)		0.931	0.848	0.694	0.569	0.536	0.566	0.910	0.587

^1^ Energy density: diets were formulated at two metabolizable energy (**ME**) levels: standard (meeting or exceeding breeder recommendations for White Leghorn hens) and low (200 kcal/kg lower than standard, achieved by substituting soybean oil with inert sand). ^2^ Corn type: diets were based on either conventional corn or Enogen corn, a genetically modified variety expressing a thermostable α-amylase (AMY797E) directly in the endosperm. No significant interactions or main effects of energy density and corn type were observed on body composition at 45 weeks of age (*p* > 0.05; n = 10). Abbreviations: BMD, bone mineral density; BMC, bone mineral content.

**Table 14:** Microarchitecture of total section of femur bone in laying hens fed conventional corn or Enogen corn diets with standard or reduced energy at 45 weeks of age.

Parameters		Total Section ^1^				
		BMD (g/cm^3^)	BMC (g)	TV (mm^3^)	BV (mm^3^)	BV/TV (%)
***Interaction effect***						
**Energy density** **^2^**	**Corn type** **^3^**					
Standard	Conventional	0.648	0.222	342	153	44.6
	Enogen	0.623	0.202	326	140	43.4
Low	Conventional	0.606	0.211	348	146	42.2
	Enogen	0.633	0.320	506	251	47.9
SEM		0.019	0.048	76	43	2.6
*p* (Interaction)		0.179	0.191	0.256	0.179	0.188
***Main effects***						
**Energy density**						
Standard		0.635	0.212	334	147	44.0
Low		0.620	0.265	427	199	45.1
**Corn type**						
Conventional		0.627	0.216	345	150	43.4
Enogen		0.628	0.261	416	195	45.7
SEM		0.013	0.034	53	30	1.8
*p* (Energy density)		0.411	0.278	0.228	0.235	0.669
*p* (Corn type)		0.960	0.364	0.353	0.297	0.379

^1^ Total section: a 7.5 mm section of the femur bone, including cortical, trabecular, and medullary regions (Figure 1). ^2^ Energy density: diets were formulated at two metabolizable energy (**ME**) levels: standard (meeting or exceeding breeder recommendations for White Leghorn hens) and low (200 kcal/kg lower than standard, achieved by substituting soybean oil with inert sand). ^3^ Corn type: diets were based on either conventional corn or Enogen corn, a genetically modified variety expressing a thermostable α-amylase (AMY797E) directly in the endosperm. No significant interactions or main effects of energy density and corn type were observed on the microarchitecture of total bone section of femur at 45 weeks of age (*p* > 0.05; n = 10). Abbreviations: BMD = bone mineral density; BMC = bone mineral content; TV = tissue volume; BV = bone volume; BV/TV = bone volume fraction.

**Table 15:** Microarchitecture of cortical section of femur bone in laying hens fed conventional corn or Enogen corn diets with standard or reduced energy at 45 weeks of age.

Parameters		Cortical Section ^1^											
		BMD (g/cm^3^)	BMC (g)	TV (mm^3^)	BV (mm^3^)	BV/TV (%)	NCP	VCP (mm^3^)	CP (%)	VOP (mm^3^)	OP (%)	VTP (mm^3^)	TP (%)
***Interaction effect***													
**Energy density ^2^**	**Corn type ^3^**												
Standard	Conventional	1.284	0.107	84	83	98.7	227	0.283	0.35	0.803	0.95	1.087	1.30
	Enogen	1.302	0.104	80	79	99.1	199	0.189	0.24	0.575	0.71	0.764	0.95
Low	Conventional	1.263	0.106	84	82	98.0	248	0.346	0.44	1.283	1.61	1.629	2.05
	Enogen	1.250	0.135	109	108	98.4	325	0.318	0.32	1.321	1.30	1.638	1.61
SEM		0.016	0.013	11	11	0.3	45	0.058	0.07	0.244	0.29	0.286	0.34
*p* (Interaction)		0.345	0.237	0.208	0.209	0.899	0.251	0.571	0.918	0.590	0.898	0.566	0.899
***Main effects***													
**Energy density**													
Standard		1.293 ^a^	0.106	82	81	98.9 ^a^	213	0.236	0.29	0.689 ^b^	0.83 ^b^	0.925 ^b^	1.12 ^b^
Low		1.257 ^b^	0.120	96	95	98.2 ^b^	287	0.332	0.38	1.302 ^a^	1.46 ^a^	1.634 ^a^	1.83 ^a^
**Corn type**													
Conventional		1.274	0.106	84	82	98.3	238	0.315	0.40	1.043	1.28	1.358	1.67
Enogen		1.276	0.120	95	93	98.7	262	0.253	0.28	0.948	1.00	1.201	1.28
SEM		0.012	0.009	8	8	0.2	32	0.041	0.05	0.173	0.20	0.202	0.24
*p* (Energy density)		0.035	0.269	0.207	0.2256	0.044	0.112	0.106	0.249	0.017	0.036	0.018	0.044
*p* (Corn type)		0.880	0.323	0.337	0.3263	0.255	0.598	0.294	0.118	0.699	0.337	0.587	0.255

^1^ Cortical section: cortical region of a 7.5 mm section of the femur bone (Figure 1). ^2^ Energy density: diets were formulated at two metabolizable energy (**ME**) levels: standard (meeting or exceeding breeder recommendations for White Leghorn hens) and low (200 kcal/kg lower than standard, achieved by substituting soybean oil with inert sand). ^3^ Corn type: diets were based on either conventional corn or Enogen corn, a genetically modified variety expressing a thermostable α-amylase (AMY797E) directly in the endosperm. ^a,b^ Values within a column with different superscripts differ significantly (*p* < 0.05; n = 10). Significant main effects of energy density were observed on bone mineral density, bone volume fraction, volume of open pores, open porosity, volume of total pores and total porosity at 45 weeks of age (*p* < 0.05). Abbreviations: BMD = bone mineral density; BMC = bone mineral content; TV = total volume; BV = bone volume; BV/TV = bone volume fraction; NCP = number of closed pores; VCP = volume of closed pores; CP = closed porosity; VOP = volume open pores; OP = open porosity; VTP = volume of total pores; TP = total porosity.

**Table 16:** Microarchitecture of trabecular section of femur bone in laying hens fed conventional corn or Enogen corn diets with standard or reduced energy at 45 weeks of age.

Parameters		Trabecular Section ^1^								
		BMD (g/cm^3^)	BMC (g)	BV (mm^3^)	Tb.Th (mm)	Tb.N (mm^−1^)	Tb.Pf (mm^−1^)	Conn.D	DA	SMI
***Interaction effect***										
**Energy density ^2^**	**Corn type ^3^**									
Standard	Conventional	1.050	0.010	9.3	0.120	8.4	5.88	61.8	2.08	1.47
	Enogen	1.048	0.007	6.8	0.119	8.4	6.64	58.6	2.10	1.58
Low	Conventional	1.052	0.009	8.4	0.120	8.4	5.76	60.5	2.15	1.44
	Enogen	1.052	0.022	20.9	0.123	8.2	6.15	60.7	1.99	1.54
SEM		0.006	0.006	5.9	0.003	0.2	0.35	6.1	0.09	0.06
*P* (Interaction)		0.886	0.207	0.209	0.463	0.535	0.598	0.784	0.350	0.920
***Main effects***										
**Energy density**										
Standard		1.049	0.008	8.1	0.120	8.4	6.26	60.2	2.09	1.53
Low		1.052	0.015	14.7	0.121	8.3	5.95	60.6	2.07	1.49
**Corn type**										
Conventional		1.051	0.009	8.9	0.120	8.4	5.82	61.2	2.12	1.46
Enogen		1.050	0.014	13.9	0.121	8.3	6.39	59.7	2.05	1.56
SEM		0.004	0.004	4.1	0.002	0.1	0.25	4.3	0.06	0.04
*p* (Energy density		0.614	0.262	0.266	0.620	0.661	0.387	0.942	0.809	0.576
*p* (Corn type)		0.877	0.399	0.398	0.751	0.804	0.109	0.808	0.448	0.094

^1^ Trabecular section: trabecular region of a 7.5 mm section of the femur bone (Figure 1). ^2^ Energy density: diets were formulated at two metabolizable energy (**ME**) levels: standard (meeting or exceeding breeder recommendations for White Leghorn hens) and low (200 kcal/kg lower than standard, achieved by substituting soybean oil with inert sand). ^3^ Corn type: diets were based on either conventional corn or Enogen corn, a genetically modified variety expressing a thermostable α-amylase (AMY797E) directly in the endosperm. No significant interactions or main effects of energy density and corn type were observed on the microarchitecture of trabecular section of femur bone at 45 weeks of age (*p* > 0.05; n = 10). Abbreviations: BMD = bone mineral density; BMC = bone mineral content; BV = bone volume; Tb.Th = trabecular thickness; Tb.N = trabecular number; Tb.Pf = trabecular pattern factor; Conn.D = connectivity density; DA = degree of anisotropy; SMI = structure model index.

**Table 17:** Microarchitecture of medullary section of femur bone in laying hens fed conventional corn or Enogen corn diets with standard or reduced energy at 45 weeks of age.

Parameters		Medullary Section ^1^				
		BMD (g/cm^3^)	BMC (g)	TV (mm^3^)	BV (mm^3^)	BV/TV (%)
***Interaction effect***						
**Energy density ^2^**	**Corn type ^3^**					
Standard	Conventional	0.367	0.082	223	36	15.93
	Enogen	0.324	0.068	214	29	14.59
Low	Conventional	0.319	0.073	229	31	13.69
	Enogen	0.321	0.118	333	83	21.71
SEM		0.025	0.024	51	20	3.16
*p* (Interaction)		0.368	0.226	0.272	0.145	0.147
***Main effects***						
**Energy density**						
Standard		0.345	0.075	218	33	15.26
Low		0.320	0.095	281	57	17.70
**Corn type**						
Conventional		0.343	0.077	226	33	14.81
Enogen		0.323	0.093	274	56	18.15
SEM		0.018	0.017	36	14	2.23
*p* (Energy density)		0.309	0.409	0.226	0.234	0.445
*p* (Corn type)		0.426	0.519	0.357	0.260	0.296

^1^ Medullary section: medullary region of a 7.5 mm section of the femur bone (Figure 1). ^2^ Energy density: diets were formulated at two metabolizable energy (**ME**) levels: standard (meeting or exceeding breeder recommendations for White Leghorn hens) and low (200 kcal/kg lower than standard, achieved by substituting soybean oil with inert sand). ^3^ Corn type: diets were based on either conventional corn or Enogen corn, a genetically modified variety expressing a thermostable α-amylase (AMY797E) directly in the endosperm. No significant interactions or main effects of energy density and corn type were observed on the microarchitecture of medullary section of femur bone at 45 weeks of age (*p* > 0.05; n = 10). Abbreviations: BMD = bone mineral density; BMC = bone mineral content; TV = total volume; BV = bone volume; BV/TV = bone volume fraction.

## Data Availability

The original contributions presented in this study are included in the article. Further inquiries can be directed to the corresponding author.
